# Implementing and evaluating group interpersonal therapy for postnatal depression in Lebanon and Kenya—individually randomised superiority trial

**DOI:** 10.1186/s13063-024-08039-3

**Published:** 2024-03-26

**Authors:** Peter Fonagy, Rabih El Chammay, Carol Ngunu, Manasi Kumar, Lena Verdeli, Elizabeth Allison, Ghida Anani, Pasco Fearon, Fouad Fouad, Zoe Hoare, Lucina Koyio, Henrietta Moore, Andrew Nyandigisi, Stephen Pilling, Hannah Sender, Jolene Skordis, Rachel Evans, Gerard Joseph Abou Jaoude, Beatrice Madeghe, Sandra Pardi Arsen Maradian, Ciara O’Donnell, Elizabeth Simes, Alexandra Truscott, Grace Nduku Wambua, Obadia Yator

**Affiliations:** 1 University College London, London, UK; 2 Anna Freud, London, UK; 3 Ministry of Public Health Lebanon, Baabda, Lebanon; 4 National Mental Health Programme, Beirut, Lebanon; 5 Nairobi City County Government, Nairobi, Kenya; 6 University of Nairobi, Nairobi, Kenya; 7 Columbia University, New York, NY USA; 8 ABAAD, Beirut, Lebanon; 9 American University of Beirut, Beirut, Lebanon; 10 Bangor University, Bangor, UK; 11 Health Strat, Nairobi, Kenya

**Keywords:** Post-natal depression, Group interpersonal therapy, Infant development, High-quality standard care, Multisite randomised controlled trial, Randomised controlled trial, Cultural adaptation, Economic evaluation, LMIC

## Abstract

**Background:**

Depression ranks as the foremost mental health concern among childbearing women. Within low- and middle-income countries (LMICs), between 20 and 25% of women encounter depression during pregnancy or soon after delivery. This condition impacts not only the mothers but also their offspring. Offspring of women suffering from postnatal depression (PND) exhibit suboptimal cognitive development and increased emotional and behavioural issues throughout their growth. This scenario becomes more pronounced in LMICs, where numerous adversities further jeopardise children’s developmental progress. Despite antenatal services providing a pivotal platform to address women’s mental health needs, PND treatment remains inaccessible in many LMICs. The World Health Organization advocates interpersonal psychotherapy (IPT) for treating depression. While research from high-income countries has established the efficacy of IPT and group-IPT (g-IPT) for PND, its effectiveness within the LMIC context and its potential benefits for child development remain uncharted. This study seeks to gauge the potency of g-IPT for women with PND in two LMICs.

**Methods:**

This multi-site randomised controlled trial is a continuation of two preceding phases—conceptual mapping and a feasibility study executed in Lebanon and Kenya. Insights gleaned from these phases underpin this comprehensive RCT, which contrasts the efficacy and cost-effectiveness of high-quality standard care (HQ-SC) augmented with g-IPT against HQ-SC in isolation. The trial, characterised as an individually randomised superiority assessment, targets women with postnatal depression in Beirut, Lebanon, and Nairobi, Kenya. It aims to determine if culturally tailored g-IPT, administered within community settings in both countries, outperforms HQ-SC in influencing child developmental outcomes, maternal depression, and the quality of the mother–child bond.

**Discussion:**

The SUMMIT trial, designed with pragmatism, possesses the magnitude to evaluate g-IPT within two LMIC frameworks. It seeks to enlighten policymakers, service commissioners, professionals, and users about g-IPT’s potential to alleviate maternal PND and bolster child developmental outcomes in LMICs. Additionally, the trial will generate valuable data on the clinical and economic merits of high-quality standard care.

**Trial registration:**

ISRCTN, ISRCTN15154316. Registered on 27 September 2023, https://doi.org/10.1186/ISRCTN15154316

**Supplementary Information:**

The online version contains supplementary material available at 10.1186/s13063-024-08039-3.

## Administrative information

Note: the numbers in curly brackets in this protocol refer to the SPIRIT checklist item numbers. The order of the items has been modified to group similar items (see http://www.equator-network.org/reporting-guidelines/spirit-2013-statement-defining-standard-protocol-items-for-clinical-trials/).

**Table:** 

Title {1}	*SPIRIT guidance: Descriptive title identifying the study design, population, interventions, and, if applicable, trial acronym.* Full title: Implementing and evaluating group interpersonal therapy for postnatal depression in Lebanon and Kenya—individually randomised superiority trial Short title: SUMMIT (SUpporting Mothers’ Mental health with Interpersonal Therapy)
Trial registration {2a and 2b}	*SPIRIT guidance: Trial identifier and registry name. If not yet registered, name of intended registry.* ISRCTN registry *Item 2b is met if the register used for registration collects all items from the World Health Organization Trial Registration Data Set.* ISRCTN reference: 15154316 https://doi.org/10.1186/ISRCTN15154316
Protocol version {3}	*SPIRIT guidance: Date and version identifier.* V1.1 23.02.2023
Funding {4}	*SPIRIT guidance: Sources and types of financial, material, and other support.* National Institute for Health Research (NIHR) Research and Innovation for Global Health Transformation (RIGHT) Programme
Author details {5a}	*SPIRIT guidance: Affiliations of protocol contributors.* Professor Peter Fonagy PFo University College London, United Kingdom and Anna Freud National Centre for Children and Families (p.fonagy@ucl.ac.uk) Dr Rabih El Chammay RC Ministry of Public Health Lebanon, Lebanon (rabih.chammay@nmhp-lb.com) Dr Carol Ngunu CN Nairobi City County Government, Kenya (ngunucarol@yahoo.com) Dr Manasi Kumar MK University of Nairobi, Kenya (manni_3in@hotmail.com) Professor Lena Verdeli LV Columbia University, United States (verdeli@exchange.tc.columbia.edu) Dr Elizabeth Allison EA University College London, United Kingdom and Anna Freud National Centre for Children and Families (e.allison@ucl.ac.uk) Professor Ghida Anani GA ABAAD, Lebanon (ghida.anani@abaadmena.org) Professor Pasco Fearon PFe University College London, United Kingdom (p.fearon@ucl.ac.uk) Dr Fouad Fouad FF American University of Beirut, Lebanon (mm157@aub.edu.lb) Dr Zoe Hoare ZH Bangor University, United Kingdom (z.hoare@bangor.ac.uk) Dr Lucina Koyio LK Nairobi City County Goverment, Kenya (koyiolucina@yahoo.co.uk) Professor Henrietta Moore HM University College London, United Kingdom (henrietta.moore@ucl.ac.uk) Dr Andrew Nyandigisi AN Health Strat, Kenya (anyandigisi@healthstrat.co.ke) Professor Stephen Pilling SP University College London, United Kingdom (s.pilling@ucl.ac.uk) Dr Hannah Sender HS University College London, United Kingdom (hannah.sender@ucl.ac.uk) Professor Jolene Skordis JS University College London, United Kingdom (j.skordis@ucl.ac.uk) Rachel Evans RE Bangor University, United Kingdom (r.evans@bangor.ac.uk) Gerard Joseph Abou Jaoude GJ, University College London, United Kingdom. (gerard.jaoude.15@ucl.ac.uk) Dr Beatrice Madeghe BM University of Nairobi, Kenya (bearecha@gmail.com) Sandra Pardi Arsen Maradian SM National Mental Health Programme, Lebanon (pardi.maradian@nmhp-lb.com) Ciara O’Donnell CO’D University College London, United Kingdom and Anna Freud National Centre for Children and Families (ciara.o'donnell.16@ucl.ac.uk) Dr Elizabeth Simes ES University College London, United Kingdom and Anna Freud National Centre for Children and Families (e.simes@ucl.ac.uk) Alexandra Truscott AT University College London, United Kingdom and Anna Freud National Centre for Children and Families (Alexandra.Truscott@annafreud.org) Dr Grace Nduku Wambua GNW University of Nairobi, Kenya (wambua.nduku@gmail.com) Dr Obadia Yator OY University of Nairobi, Kenya (obadiayator@gmail.com)
Name and contact information for the trial sponsor {5b}	*SPIRIT guidance: Name and contact information for the trial sponsor.* Sponsor Representative: Professor Alan Thompson Dean’s Office Faculty of Brain Sciences Maple House, 149 Tottenham Court Road London W1T 7NF alan.thompson@ucl.ac.uk
Role of sponsor {5c}	*SPIRIT guidance: Role of study sponsor and funders, if any, in study design; collection, management, analysis, and interpretation of data; writing of the report; and the decision to submit the report for publication, including whether they will have ultimate authority over any of these activities.* Neither the funder nor sponsor have any role in study design, collection, management, analysis, interpretation of data, writing reports or decisions to submit the report for publication.

## Introduction

### Background and rationale {6a}

Post-natal depression (PND) stands as a predominant mental health condition, posing a global challenge to both maternal health and child development, with a pronounced impact in low- and middle-income countries (LMICs) [1, 2]. Depression represents the most significant portion of the mental disorder burden among childbearing women globally [3]. Contemporary pooled prevalence estimates pinpoint that 19.2% of women in low-income countries and 18.7% in middle-income nations grapple with major depression during pregnancy and postpartum, figures nearly twice as high as those in high-income countries (HICs) [4]. Nationally, PND prevalence exhibits a distinct socio-economic gradient. PND rates surge in the context of heightened socio-economic deprivation, gender-based violence, and emotional maltreatment and in settings marked by humanitarian crises [5].

Beyond the suffering and disability endured by women with PND, ample evidence underscores that their children are predisposed to significant cognitive impairments and compromised mental and physical well-being [1, 2, 6]. These suboptimal developmental outcomes for children are attributed to the deterioration in the quality and sensitivity of mother-infant interactions linked to the mother’s PND [7, 8]. The enduring adverse effects of maternal depression on child development are particularly pronounced when coupled with other risk elements like poverty, limited parental education, and coexistent maternal physical and psychological disorders [9–11]. These risk elements are more prevalent in LMICs. Consequently, addressing maternal PND emerges as a pressing public health concern and a principal focus for policymakers. Their aim is to improve both maternal well-being and early childhood development, striving to diminish health disparities in alignment with the directives of the Sustainable Development Goals [12].

Effective intervention for PND can mitigate the detrimental effects of poverty on child development, given its clear characterisation as a modifiable risk factor often recognised during periods of heightened health service engagement, even in resource-constrained environments [13]. For numerous women in LMICs, antenatal and immediate postnatal periods represent unique encounters with healthcare, presenting a pivotal opportunity to diagnose and manage maternal mental health issues and bolster early child development [14]. Yet, notwithstanding the profound consequences of PND for maternal and child health outcomes, and the significant potential gains of effective interventions, there remains a conspicuous paucity of dedicated research and scant formal health service allocation. This dearth is discernible even in HICs, including the UK [15], but the discrepancy is most pronounced in LMICs [14, 16, 17].

A robust body of evidence endorses the utility of psychological therapies for treating adult depression, with several interventions demonstrating moderate to large treatment effects when compared to waitlist controls [18, 19]. Informed by this evidence and its literature review, the UK’s National Institute for Clinical Excellence (NICE) advocates for cognitive behavioural therapy, interpersonal therapy, behavioural activation, and behavioural couples therapy, alongside antidepressant medication, for adults experiencing moderate to severe depression [20]. Similarly, the World Health Organization’s (WHO) mhGAP programme, dedicated to reviewing and disseminating evidence-driven mental health interventions in resource-limited settings, endorses cognitive-behavioural therapy (CBT), interpersonal therapy (IPT), and behavioural activation (BA) [21].

Group interpersonal therapy (g-IPT) emerges as a particularly promising modality for addressing postnatal depression within an LMIC milieu. The WHO champions g-IPT as a primary treatment for depression, and its mhGAP initiative incorporates an 8-session group framework deployable by supervised facilitators lacking prior mental health expertise [22]. A randomised controlled study led by Bolton et al. [23] evidenced that g-IPT, when administered by trained local residents, markedly ameliorated depression and functional deficits in a rural Ugandan setting. A Kenyan-based open trial reflected that g-IPT, facilitated by comparably trained personnel, was deemed acceptable, feasible, and induced significant symptomatic and functional enhancements [24]. Qualitative studies have provided support for the experienced relevance of the IPT framework in Kenya and Uganda [25, 26]. In Lebanon, in collaboration with the Lebanese Ministry of Public Health, clinicians have prioritised g-IPT for PND as a key training domain, with capacity-building initiatives for individual g-IPT delivery in progress. Notably, while g-IPT has not been extensively employed for PND within LMICs, a 2018 meta-analysis encompassing 28 studies from high-income countries (HICs) ascertained that g-IPT consistently ameliorates depression, anxiety, and interpersonal dynamics in perinatal women, exhibiting notable effect magnitudes [10].

Several investigations have tailored g-IPT to cater to socio-economically marginalised women, demonstrating its aptness for low-income women facing treatment accessibility challenges [27]. The relational emphasis of g-IPT is especially pertinent during the transitional phase of motherhood [10], offering a structured array of therapeutic interventions that address depressive symptoms while concurrently supporting the mother–child relationship [28]. The forthcoming trial proposes a culturally adapted g-IPT for PND, building upon prior research and the study team’s preliminary efforts in northern Uganda. Here, g-IPT has been adapted for a financially constrained rural cohort of women with infants and has been incorporated into an NGO-driven nutrition initiative to counteract stunting. This foundation also exploits insights from feasibility studies executed in Nairobi and Beirut. The preliminary stages of the present research initiative have harnessed collaborative endeavours to construct contextually appropriate postnatal mental health care models for depression, delineating locally pertinent care pathways for screening, treatment, and risk management. This has facilitated the culturally sensitive adaptation of g-IPT. The subsequent randomised controlled trial is poised to evaluate the modified g-IPT’s efficacy in enhancing maternal depression and child developmental outcomes.

Although there is compelling evidence validating the effectiveness of psychological therapies like g-IPT in managing PND, and robust models are available for tailoring such interventions to resource-limited settings, the proof of their capacity to boost child outcomes by mitigating the impact of the child’s exposure to maternal depression remains inconclusive, primarily due to insufficiently powered investigations. Cuijpers and co-authors [29] concluded that addressing maternal PND via psychological therapy results in enhanced child outcomes, registering an average effect size of *d* = 0.40. However, only five studies (all originating from HICs) contributed to this effect estimate, characterised by their limited scale (each having group sizes of *N* < 50) and diverse outcomes and intervention methodologies. The relatively expansive trial orchestrated by Rahman et al., which was not initially conceptualised to gauge treatment effects on child cognitive or emotional evolution, subsequently reported no enduring advantages for child outcomes resulting from PND intervention, despite a pronounced positive effect on maternal mental health [30, 31]. However, the extended interval between treatment completion and this subsequent evaluation at age 7 circumscribes the inferences that might be extrapolated from this investigation.

Given the substantial evidence linking PND to detrimental effects on child development, especially in LMIC settings, coupled with the paucity and variable quality of evidence on the efficacy of depression treatments in offsetting these impacts, there is an urgent need for well-controlled trials in resource-limited contexts. To drive advances in this field, it is imperative that subsequent PND trials are conducted in LMIC settings, integrated within sustainable healthcare infrastructures. Furthermore, such trials should be methodically designed to possess sufficient power to discern realistic downstream effects on child outcomes (e.g. *d* = 0.40) and should employ rigorous—and, wherever feasible, objective—means of assessing children’s developmental outcomes. The current trial is part of a larger research programme that has involved conceptual mapping work to enhance understanding of how PND is experienced and thought about in both Lebanon and Kenya, as well as a feasibility trial. The feasibility trial, which concluded in November 2022, recruited 87 participants in Kenya and 35 in Lebanon, and randomised them into either the intervention (High-Quality Standard Care plus g-IPT) or control arm High-Quality Standard Care (HQ-SC only), allowing us to test the randomisation system for this RCT. Both interventions were delivered and participants were followed up for 24 weeks. This RCT will draw on the lessons learned during the feasibility phase and will expand the capacity to deliver a full trial.

## Objectives {7}

The primary objective of SUMMIT is to investigate the following research question: Does culturally adapted group interpersonal therapy (g-IPT) for women with postnatal depression (PND) in community or primary care settings in Kenya and Lebanon yield superior outcomes in terms of child development, maternal depression, and the mother–child relationship compared to high-quality standard care (HQ-SC) alone?

Furthermore, SUMMIT will:

1. Conduct cost and cost-effectiveness analyses for both g-IPT and HQ-SC in both locales and determine the incremental cost-effectiveness of g-IPT in relation to HQ-SC alone.
2. Endeavour to strengthen local clinical and research capabilities by:
  - Developing integrated care pathways to facilitate identification and treatment (either g-IPT or HQ-SC) of women with PND.
  - Training local practitioners in the delivery of the adapted g-IPT model.
  - Educating local researchers in participant recruitment and administration of robust outcome measures.
  - Aspire to refine local training, supervisory techniques, and treatment monitoring strategies to ensure the sustainability and transportability of the adapted models.

## Trial design {8}

This study will implement an individually randomised controlled superiority trial comparing HQ-SC combined with culturally adapted g-IPT against HQ-SC alone for women diagnosed with postnatal depression in Beirut, Lebanon, and Nairobi, Kenya. Eligible mothers will undergo a baseline assessment and will then be randomised, on a 1.33:1 allocation ratio, to one of the two intervention arms: HQ-SC combined with g-IPT or HQ-SC alone. Follow-up assessments for participants across both intervention arms will be conducted by the research teams at intervals of 8, 13, 24, 36, and 52 weeks subsequent to the initial clinical contact. An illustrative diagram detailing the overall trial flow can be found in Additional file 1.

## Methods: participants, interventions, and outcomes

### Study setting {9}

Participants will be identified from a range of healthcare settings in Beirut, Lebanon, and Nairobi, Kenya, including Maternal and Child Health clinics in primary care services, private clinics, NGOs, and other healthcare establishments. The specific settings will vary between the two countries.

*Kenya*: Most Kenyans access healthcare through the public sector, which is structured across six service levels, from level 1 to level 6 hospitals. For this study, the focus will be on Nairobi County, with participants being recruited from the following locations: Huruma Lions Health Centre, Riruta Health Centre, Githurai Health Centre, Kangemi Health Centre, Mbagathi Hospital, and Mathari Teaching and Referral Hospital. These sites represent a combination of public community primary care facilities and hospitals.

*Lebanon*: In Lebanon, all recruitment venues are primary healthcare centres (PHCs) affiliated with the Ministry of Public Health (MOPH) network. All participating sites are located within the Beirut region. They include Makassed Primary Healthcare Centre, Howard Karagheusian Commemorative Center, Dar Al Fatwa Primary Healthcare Centre, Dar El Hawraa Primary Healthcare Centre, and Hariri Foundation Primary Healthcare Centre.

### Eligibility criteria {10}

The PHQ-9 [32] will be employed to identify potential participants for the study among women attending Maternal and Child Health clinics in primary care services, private clinics, NGOs, and other healthcare settings. We aim to recruit mothers with infants aged between 6 and 35 weeks. This specific developmental window allows us to use a consistent set of well-validated measures for this rapidly developing group of children. Additionally, it reflects the peak child age during which PND is most likely to present to services. Clinicians will pinpoint service users within their caseload who align with the inclusion and exclusion criteria and will collaborate with researchers to initiate contact. The participant selection process will vary slightly between countries, elaborated further below.

*Kenya*: In Nairobi, where universal healthcare is available, there is a notable uptake of public postnatal health checks, primarily for immunisation and screening by community health workers. These workers will receive training to administer the Whooley Questions [33] as an introductory screen for mothers. Those mothers who yield positive results on the Whooley Questions (by answering “yes” to one or both questions) will proceed to complete the PHQ-9. With its proven sensitivity and specificity in detecting depression, mothers who score positively on the PHQ-9 will be asked for their consent and subsequently referred for a research assessment.

*Lebanon*: In Beirut, clinical staff across a spectrum of public and private primary healthcare services that offer immunisation and postnatal care will be trained to administer the PHQ-9 and to refer cases that screen positively into the study.

Eligibility for participation will require all candidates to satisfy the following inclusion criteria:

- Be at least 18 years of age
- Identify as female
- Display indications of postnatal depression as represented by a score of 12 or higher on the PHQ-9 at the initial assessment
- Be a mother of an infant aged between 6 and 35 weeks at the screening time

The study will exclude participants based on the following criteria:

- Mothers diagnosed with psychotic conditions, such as bipolar disorder, anorexia nervosa, or substance dependency
- Mothers whose infants have severe physical health or neurodevelopmental issues.

### Who will take informed consent? {26a}

A designated member of the research team will describe the trial’s objectives, methodologies, expected benefits, and potential risks to the prospective participants. Following this, they will obtain written informed consent before initiating any trial-related procedures. To ensure participants have ample time to reflect on their decision, they will be provided a minimum of 24 h after receiving this information to consider their participation. Only trained and competent research assistants, specifically authorised for this task, will oversee the consent process. The consent form and the Patient Information Sheet (PIS) will be available in the participant’s native language (e.g. English, Swahili, and Arabic).

Participants will be assured of their rights before signing the consent form. They will be informed that:

- They can pose any questions concerning the information sheet or the consent form at any juncture.
- Their participation is entirely voluntary, with no obligations attached.
- They retain the right to withdraw from the trial at any stage without needing to specify any reasons.

No trial-related activities will commence until the participant affirms their consent by signing the consent form. However, signing this consent does not automatically imply their enrolment in the trial. Participants will be provided a copy of the signed Informed Consent form for their records, while the original will be safeguarded in the trial file at the respective local site.

To guarantee genuine and informed consent, the research team will take measures to confirm participants fully grasp the provided information about the trial and the expectations from them. The consent process will be conducted in the participant’s native language to ensure clarity and comprehension. The PIS and consent form will be periodically reviewed and revised as required throughout the trial, such as when new safety data emerges. If changes are significant, participants will be approached for re-consent accordingly.

### Interventions

#### Explanation for the choice of comparators {6b}

Group interpersonal psychotherapy (g-IPT) is a talk therapy developed in the 1970s as a structured and time-bound treatment for adults with depression. Rooted in the interpersonal psychoanalysis of Sullivan [34] and the attachment theory of Bowlby [35, 36], it places a significant emphasis on interpersonal relationships, life transitions, and therapeutic alliance. The therapy can be delivered both individually or in groups, as in g-IPT [37]. Originally, the treatment was designed to address depressive symptoms, lessen interpersonal conflicts, and enhance interpersonal skills. To date, g-IPT has been recognised as an effective remedy for various mental health disorders and is also deemed valuable as a preventive intervention. Central to g-IPT is the notion that interpersonal adversities, such as grief, disputes, role transitions, loneliness, and social isolation, can instigate and exacerbate depression. The therapy strives for recovery from the current depressive episode by shedding light on the correlation between the onset of depressive symptoms and interpersonal problems, coupled with skill-building to manage these issues more effectively. Given g-IPT’s emphasis on interpersonal role changes, disputes, isolation, and diminished social support, it is particularly pertinent to challenges women might encounter during and post-pregnancy [10].

For this study, HQ-SC has been selected as the control treatment. Based on the WHO’s stress management guide titled “Doing What Matters in Times of Stress” [38], HQ-SC is a guided psychoeducation intervention administered over two sessions. Ethical standards mandate that once women diagnosed with PND are pinpointed, they should receive some form of support. For our study, it was imperative to opt for a control treatment consistent across both countries, irrespective of differing cultural backgrounds. The conventional treatment (TAU) for PND in both Kenya and Lebanon can diverge in terms of accessibility and modes of delivery.

#### Intervention description {11a}

After completing the baseline outcome measures, participants will be randomly assigned to either the intervention arm (HQ-SC plus g-IPT) or the control arm (HQ-SC). All trial participants will receive HQ-SC. Those assigned to the intervention arm will subsequently receive g-IPT.

*High-quality standard care*: In both Kenya and Lebanon, high-quality standard care (HQ-SC), known as “Doing What Matters in Times of Stress” [38], teaches participants how to use a stress management guide over the course of two group sessions, with each taking between 60 and 90 min. This guide aids them in developing practical skills to handle stress effectively. Grounded in Acceptance and Commitment Therapy (ACT) [39], it is designed for anyone over 18 years of age, with its brief nature allowing for quick reading. This intervention will be extended to all women in the trial, regardless of other available interventions in the pathway of standard care.

*Group interpersonal psychotherapy* (g-IPT): g-IPT is an evidence-backed, time-limited psychotherapy tailored for depressive disorders, encompassing post-natal depression. It has been practised and validated in LMICs by professionals and trained laypersons [40]. The therapy’s goal is to address interpersonal triggers of depressive episodes, segmented into four problem areas: grief, disputes, life changes, and loneliness/social isolation [41]. The World Health Organization (WHO) has globally disseminated group IPT through a nine-session protocol (1 individual and 8 group sessions), which is employed in this study [22]. These g-IPT sessions typically last 1.5 h, except for the initial individual session (up to 2 h) and the concluding session (2–3 h). These sessions are held weekly and consist of groups of 6–10 women.

*Adaptation of g-IPT*: IPT had been previously adapted to the Kenyan and Lebanese contexts for cultural and situational relevance in earlier studies [42–44]. However, further refinements have been made for this specific study population and regional contexts. These adaptations in content and delivery of g-IPT contemplate each location’s cultural norms for young mothers related to intimacy, authority, daily routines, interactions, child-rearing practices, and communication standards (like expressions of closeness, including mother–child attachment, aggression, assertiveness, etc.). Also considered are the current societal, familial, and individual stressors arising from social instability, increased poverty, and scarce resources, which are prevalent in both regions, albeit in varied forms. Notably, issues like domestic violence and grave livelihood challenges are anticipated to be pertinent for both nations. These adaptations were meticulously documented during the pre-trial and feasibility stages, employing the Framework for Modification and Adaptations-Enhanced [45]. Feedback from participants in the feasibility trial was integrated iteratively, refining the g-IPT model, which culminated in the final clinical protocol to be assessed in the RCT.

*g-IPT training*: For the sake of sustainability and scalability, the qualifications of g-IPT providers will differ between the two study sites. In Lebanon, in line with recent national regulations for accredited psychotherapy provision, trial providers will be licensed psychologists associated with primary care locations, with one facilitator per group. In contrast, Kenya, which has adopted a task-shifting approach to bolster capacity in primary care [46], will utilise community health workers, assigning two facilitators per group. Supervisors across both sites will be professionals previously trained in IPT, such as psychologists, psychiatrists, and social workers. The training and implementation of the intervention will be overseen by two US-based g-IPT master trainers.

We will adopt the apprenticeship model of psychotherapy training, which entails a three-tiered system for sustainable capacity building: master trainers, local supervisors, and g-IPT providers [47, 48]. The master trainers, aided by local supervisors, will conduct a 4-day didactic g-IPT workshop for facilitators. This will be followed by a standardised g-IPT knowledge test with a set “pass” threshold. Facilitators who pass this test will subsequently receive supervision in weekly meetings (either onsite or online) for two groups per facilitator. These sessions will be led by the local supervisors, with occasional assistance from the master trainers. Facilitators’ competence will be gauged using a g-IPT fidelity checklist during these supervision sessions. Only facilitators who achieve a mean score per group that meets or exceeds 75% of the “adequately competent” benchmark at designated intervals (one during the pre-group phase, one at the outset, two mid-way, and one at the end) will be certified to conduct the study. Following the completion of their inaugural training case, trainees will attend a 2-day advanced workshop. Orchestrated by master trainers and local supervisors, this workshop will fortify both knowledge and expertise.

Priority will be given to supporting both facilitators and supervisors, especially considering they are contending with persistent socioeconomic challenges in both regions. A communal spirit of mutual support and collaboration will be cultivated via group supervision and inter-site conferences. These gatherings will facilitate the exchange of experiences, ideas, and assistance in applying the IPT model.

#### Criteria for discontinuing or modifying allocated interventions {11b}

By agreeing to participate in the trial, participants are giving their consent for intervention, assessments, follow-up, and data collection. However, participants retain the right to withdraw from the study at any point, without the obligation to provide a reason. The study also reserves the right to withdraw participants from treatment under certain circumstances, such as if a designated clinician deems it necessary. Such a decision may arise if there is a notable worsening of symptoms or if the participant develops a new condition that necessitates a different treatment delivered individually. For example, if a participant reports worsening symptoms such as suicidal ideation during their participation in the trial they will be referred to a clinician for review and may be offered additional talking therapy or medication if deemed appropriate.

#### Strategies to improve adherence to interventions {11c}

In ensuring the integrity of this study, we adhere to the fidelity domains outlined by the NIH Behavior Change Consortium [49]. For the study design, our approach is rooted in the interpersonal theory of depression. Both g-IPT and HQ-SC are meticulously standardised in terms of their duration and frequency. We have designed the recruitment and delivery process to avoid any contamination, and any implementation challenges arising will be reviewed in regular weekly meetings that include representatives from the study sites, the training team, and University College London (UCL).

g-IPT facilitators are considered competently trained once they achieve predetermined benchmarks. These include attending at least 80% of the training workshop and supervision sessions, scoring 70% or above in the knowledge test, and reaching a 75% benchmark in g-IPT fidelity on the checklists. The delivery of g-IPT is firmly based on a manual. We are open to potential content adaptations, provided they remain true to the original model. Before launching the RCT, any such amendments will be incorporated into the manual. Our monitoring efforts during the implementation phase include offering consistent feedback to facilitators and selecting a random subset for competence evaluation to ascertain their adherence to the model. Supervisors will oversee these evaluations, with oversight from the master trainers. We aim to evaluate the fidelity of approximately 22% of all sessions through a randomised sampling approach.

Our approach aims to enhance participant engagement from the outset, starting with the pre-group meeting. Our goal is to ensure that every participant not only receives but fully understands the g-IPT skills. We will keep a close eye on session attendance and promptly address any signs that a participant might be at risk of dropping out. The comprehension of participants will be consistently assessed during both onsite and online supervisions, wherein facilitators will elaborate on how they communicated the concepts and how participants responded. Finally, to monitor the enactment of treatment skills, we will observe and bolster participants’ ability to apply g-IPT skills in their day-to-day lives. This will be achieved through regular discussions centred on participants’ practice between sessions, often referred to as “homework”.

#### Relevant concomitant care permitted or prohibited during the trial {11d}

In line with the pragmatic nature of this trial, concomitant care and interventions are permitted as part of the SUMMIT study, based on the judgement of a designated clinician. This encompasses both medications and various forms of therapy. The singular exception is that participants in the HQ-SC care group are prohibited from receiving g-IPT.

#### Provisions for post-trial care {30}

Upon trial conclusion, participants will continue to be monitored by the local research teams for a duration of 1 year from their initial clinical contact. If any concerns arise regarding a participant’s safety during this period, the research teams are equipped and obliged to adhere to the trial’s safeguarding protocol, as well as the established protocol for reporting adverse events.

### Outcomes {12}

#### Primary outcome measure

The primary outcome is the child’s cognitive development at the 52-week (T6) follow-up, measured using the Malawi Developmental Assessment Tool (MDAT) [50]. The MDAT is a widely accepted tool for assessing children’s cognitive development. It has been employed in numerous global health studies focusing on early child development and has demonstrated sensitivity to interventions in various trials.

#### Secondary outcome measures

Secondary outcomes encompass a broad array of metrics:

- Maternal depression severity, which will be gauged using the Patient Health Questionnaire—depression module (PHQ-9) [32] at multiple time points: baseline (T1), 8 weeks (T2), 13 weeks (T3), 24 weeks (T4), 36 weeks (T5), and 52 weeks (T6).
- Family circumstances will be evaluated using the family circumstances questionnaire at T1, T3, and T6.
- The Family Care Indicator (FCI) [51] will be used to measure maternal sensitivity and indicators of family care at T1, T2, T3, T4, T5, and T6.
- Early childhood development will be assessed using the Caregiver Reported Early Development Index (CREDI) long form [52] at T1, T3, and T5.
- Anxiety levels will be gauged using the General Anxiety Disorder-7 (GAD-7) [53] at T1, T2, T3, T4, T5, and T6.
- Sleep patterns of the mother will be assessed using the Sleep Condition Indicator (SCI) [54], while the infant’s sleep will be evaluated using the Brief Infant Sleep Questionnaire—Revised Short form (BISQ) [55]. Both measures will be taken at T1, T2, T3, T4, T5, and T6.
- The infant’s physical health will be gauged using a dedicated physical health questionnaire, and their nutritional intake will be assessed using the breastfeeding outcome measure at T1, T2, T3, T4, T5, and T6.
- Social isolation and relationship satisfaction will be evaluated using the Lubben Social Network Scale (LSNS-6) [56] and the Couple Satisfaction Index (CSI-4) [57], respectively, at T1, T3, and T6.
- Mother’s health and quality of life will be assessed using the EQ-5D-5L [58] and the ICEpop CAPability measure for Adults (ICECAP-A) [59] at T1, T3, T4, and T6.
- The economic evaluation of the intervention will be determined using the SUMMIT patient cost questionnaire [60] at T3. Additionally, household economic status will be gauged using a Household Economic questionnaire at T1, and household shocks will be assessed based on the Malawi Integrated Household Survey (IHS) [61] at T3.
- Demographics, including socio-economic status, maternal education, maternal parity, and teen parent status, will be collected using the SUMMIT demographic questionnaire at T1.
- To further examine the mother’s evolving perceptions or attitudes towards post-natal depression, brief semi-structured qualitative interviews will be conducted at T1, T3, and T6.

### Participant timeline {13}

See Fig. 1.

**Fig. 1:**
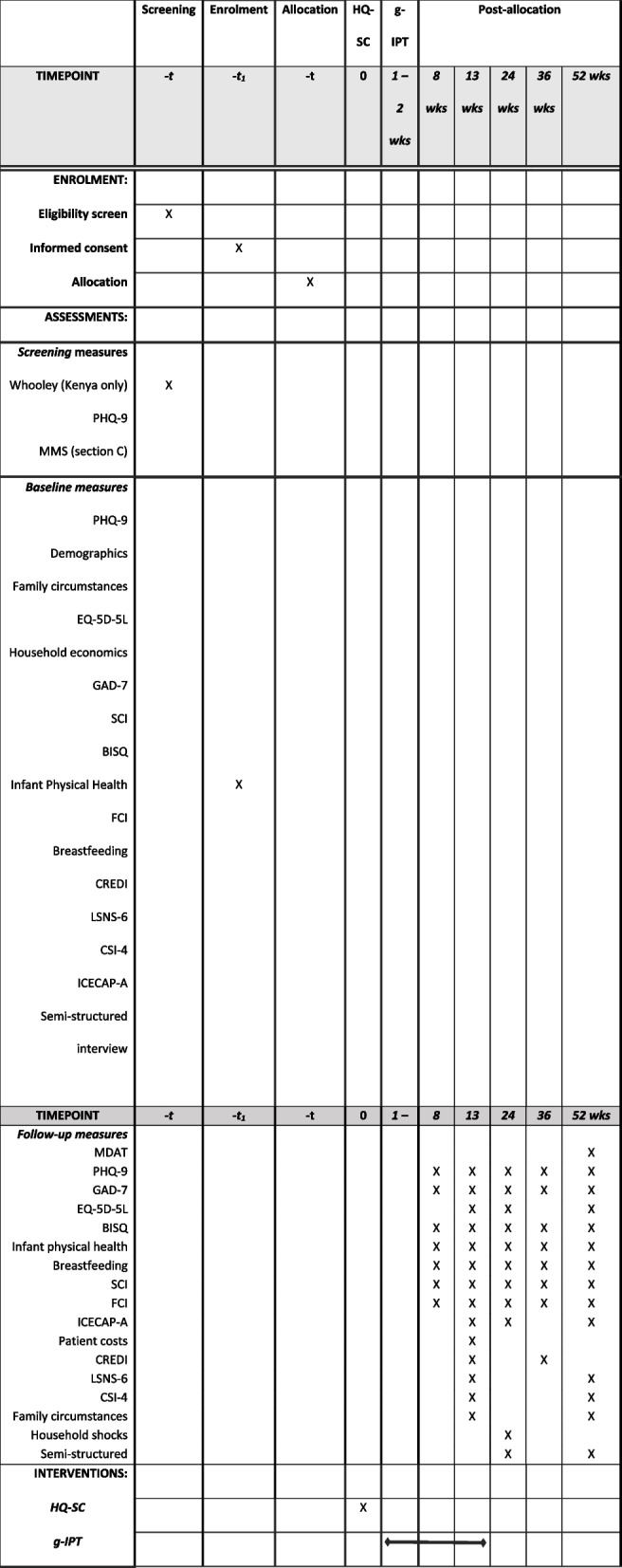
Schedule of enrolment, interventions, and assessments

### Sample size {14}

Power calculations indicate that a sample of 412 participants (*N* = 224 in the g-IPT arm and* N* = 188 in the control arm) offers 90% power to detect a standardised mean difference of 0.40 on the primary outcome, allowing for up to 25% attrition. These calculations are based on a predetermined effect size of 0.4, as informed by the Cuijpers et al. meta-analysis [29], on the primary outcome [62]. They also consider a two-tailed alpha level of 0.05 and account for clustering with an intra-class correlation (ICC) of 0.04 [63] in the treatment arm, further adjusted for an expected attrition rate of 25% [64]. The sample size estimates were derived using the “clsampsi” routine in STATA, with the assumption of a g-IPT group size of 8.

### Recruitment {15}

Four hundred twelve participants will be recruited over a 10-month period in both sites, at approximately 22 participants per month per site. Study teams will aim to recruit 6 participants per week to enable a clinical group to be established.

## Assignment of interventions: allocation

### Sequence generation {16a}

Participants will be randomised to either the control group (high-quality standard care) or the intervention group (high-quality standard care plus g-IPT). The randomisation process will be facilitated through a secure, 24-h accessible web-based platform developed and maintained by NWORTH [65]. Within the platform’s algorithm, the likelihood of a participant being allocated to a specific treatment group is dynamically recalculated based on the participants previously recruited and allocated [65]. This recalibration considers the overall allocation, stratification variables (such as participant age and recruitment site), and within each stratum level (comprising relevant combinations of stratification levels). By constantly recalculating, the algorithm ensures balance is maintained within acceptable limits of the designated allocation ratio, all while ensuring unpredictability in the randomisation process.

### Concealment mechanism {16b}

Local trial coordinators will oversee the randomisation of participants. Once randomised, participants will be informed of their group assignment. However, apart from the local trial coordinators, no other members of the local research teams will be privy to the participants’ group allocation.

### Implementation {16c}

Local trial coordinators will handle the randomisation of participants via a secure, 24-h accessible web-based platform, developed and maintained by NWORTH [65]. In the event of a system malfunction, an emergency randomisation protocol will be activated.

## Assignment of interventions: blinding

### Who will be blinded {17a}

The trial is designed as a single-blind study. All data collectors and principal investigators will remain unaware of the randomisation allocation. The randomisation of participants will be conducted off-site before the onset of the intervention. Given the unequal allocation ratio, the trial statistician will not be blinded throughout the study’s duration. However, the principal statistician (NWORTH co-applicant) who will oversee and approve all analyses will remain blind. Both the central trial coordinator and the local trial coordinators will remain unblinded to effectively oversee the randomisation process, ensuring ethical and appropriate participant allocation with minimal bias.

## Data collection and management

### Plans for assessment and collection of outcomes {18a}

Participants will be invited to complete questionnaires and participate in interviews that explore their personal history, parenting behaviours, health, and interpersonal relationships. Additionally, they will be prompted to answer questions about the health and welfare of their child. Before participants begin the questionnaires, the content will be summarised for them, and they will be reminded of their right to withdraw. This ensures that individuals who might find certain topics challenging or triggering are forewarned and can choose not to participate.

Many of the questionnaires and interview questions have their roots in clinical contexts, ensuring their potential emotional impact on respondents has been thoroughly assessed. The majority of our outcome measures have been validated for use in non-English languages. All questionnaires and interviews are conducted by a local researcher who is fluent in the language spoken by that participant.

The Research Assistants (RAs) tasked with data collection will undergo comprehensive training. This will cover the entirety of the RCT measures pack, as well as guidelines on engaging with participants, conducting interviews, and inputting data. For this particular study, we will employ live data collection methods. Specifically, outcome measures will be input directly into the REDCap database via the REDCap mobile app. REDCap (Research Electronic Data Capture) is a secure, web-based software platform designed to support data capture for research studies, providing (1) an intuitive interface for validated data capture, (2) audit trails for tracking data manipulation and export procedures, (3) automated export procedures for seamless data downloads to common statistical packages, and (4) procedures for data integration and interoperability with external sources [66, 67]. The REDCap database will be hosted by UCL.

### Plans to promote participant retention and complete follow-up {18b}

To enhance participant retention, our local teams will endeavour to gather multiple contact numbers for each participant. For those who do not possess a personal phone, we will collect contact numbers of partners, family members, or even neighbours to maintain an open line of communication throughout the study. In Kenya, our study team will leverage the aid of Community Health Volunteers (CHVs) to assist with participant engagement. These CHVs will prove invaluable in locating participants, especially in instances where the research team faces difficulties in reaching them by phone. As the CHVs maintain affiliations with both health facilities and the communities where the study is to be conducted, they offer a unique and effective bridge. To further promote retention, our local teams will liaise with participants to ensure that follow-up visits are scheduled with the utmost flexibility in mind.

Should a participant miss any or all of the intervention sessions (be it HQ-SC or g-IPT), our research team will still conduct follow-up. In such instances, we will proceed to collect all outcome measures, unless, of course, the participant expresses a desire to withdraw from the study.

### Data management {19}

The study will be conducted in strict accordance with the General Data Protection Regulations (GDPR), Good Clinical Practice (GCP), and relevant Standard Operating Procedures (SoPs). When data are stored locally at individual sites, adherence to the respective University’s SoPs will be ensured. Paramount importance will be given to patient confidentiality. All data will be securely stored on password-protected PCs/laptops, with any paper records safeguarded in locked drawers or filing cabinets within secure premises. Participants’ personal details will be coded and anonymised at the source. Each participant will be assigned a unique study number, which will be consistently utilised in all study-related documentation. The sole personal data retained throughout the study will encompass the consent form and contact details. Apart from the Consent Forms, participants’ names will not feature in any study documents, and no identifiable data will be transmitted outside the host nation. Clinical data will be archived using the participant’s unique study number, ensuring that paper records of the consent form are stored separately from this clinical data.

Quantitative data will be pseudonymised and entered directly into the central REDCap database—a secure, cloud-based platform—using only the participant’s unique study number. Local data collectors will utilise the REDCap app [66, 67], installed on a password-secured tablet, for data collection purposes. They will be thoroughly trained in its usage, complemented by a user manual for reference. The key linking ID codes and personal identifiers will remain securely with the site teams in Kenya and Lebanon, ensuring they remain undisclosed to teams in the UK or USA. All research participants’ personal data will be stored at each research site in password-protected files, exclusively accessible to the research team members.

Participants will be informed of who will have access to their records, along with the specific purposes, as outlined in the information sheet. Only members of the research team will be granted data access. Authorised individuals from the research team and regulatory bodies in both Lebanon and Kenya might review case notes. This access is explicitly specified in the consent form. Training sessions, encompassing GCP and GDPR, are provided to staff at the onset of the trial. In alignment with the GDPR 2018 stipulations, participants will receive detailed explanations regarding the handling of their data and the legal rationale for processing their personal details. The trial will adhere to the Kenya government Data Protection Act 2019, Article 31(c) and (d) of the Kenyan constitution, and the policy of the National Mental Health Programme in Lebanon, ensuring GDPR compliance in Kenya.

### Confidentiality {27}

All data will be processed in alignment with the UK Data Protection Act 1998.

Electronic and paper case report forms (CRFs) will not contain the participant’s name or any personally identifiable information. Instead, the participant’s trial identification number will serve for identification purposes. This procedure will be elucidated in the Patient Information Sheet, and participants’ consent regarding this approach will be obtained.

## Statistical methods

### Statistical methods for primary and secondary outcomes {20a}

All quantitative analyses will be conducted on an intention-to-treat (ITT) basis at the NWORTH Clinical Trials Unit. A comprehensive statistical analysis plan will be drafted and approved by the trial team before data collection concludes and will be open for review by independent committees.

Baseline characteristics will be summarised both overall and by treatment group (intervention and control), as well as by site subgroup. Descriptive statistics for all outcome variables will be generated at every time point.

Categorical variables will be showcased with counts and percentages. Continuous variables will be represented using means, standard deviations, and ranges. For non-normally distributed continuous variables, medians and interquartile ranges will be used.

The CONSORT details will be furnished with data on participant numbers.

The primary analysis will evaluate MDAT scores at 52 weeks post-initial clinical contact. A general linear mixed model will be employed, which accounts for clustering in the intervention arm. This model will adjust for allocation group and study centre. Additional covariates or factors to be integrated into the model will be pre-determined in the SAP. All statistical tests will be two-sided and paired with 95% confidence intervals.

Analyses will be executed in STATA or R, adhering to the ITT principle. The significance level (alpha) will be set at 5% (two-tailed). Linear mixed modelling, taking into account clustering by g-IPT group in the treatment arm, will be used, controlling for stratification factors (site and age group). It will also account for pre-defined covariates and model the longitudinal effects of time, as well as any pertinent time-treatment interactions.

Secondary outcomes will be evaluated using a comparable model suitable for the outcome type and will factor in baseline measures where relevant, in addition to stratification and pre-defined covariates.

### Interim analyses {21b}

There is no planned interim analysis for the study.

### Methods for additional analyses (e.g. subgroup analyses) {20b}

The Statistical Analysis Plan will detail all intended supplementary analyses.

*Health economics*: An economic evaluation of group interpersonal psychotherapy (g-IPT) versus high-quality standard care (HQ-SC) will be undertaken from both provider and participant perspectives. Participant costs will encompass travel, care-seeking, and opportunity costs. Provider costs will be categorised into set-up and implementation and will be approximated using a combination of bottom-up and top-down methodologies. Both financial and economic costs will be determined by considering indirect costs such as volunteer time and donated goods. The results will display the average cost per participant for both g-IPT and HQ-SC from the viewpoints of the provider, participant, and society (combining both provider and participant). The financial expenditure of implementation will estimate the total amount necessary to provide HQ-SC and g-IPT on a larger scale. All outcomes will be delineated separately for Kenya and Lebanon in both local currencies and international dollars.

These costs will be amalgamated with trial outcomes to compute the cost-effectiveness of g-IPT in comparison to HQ-SC. The primary analysis will have a time horizon of 52 weeks, consistent with the trial’s follow-up duration. Net differences in expenses between g-IPT and HQ-SC will be juxtaposed with net differences in outcomes to compute the incremental cost-effectiveness ratios (ICERs). ICER computations will be rooted in primary (PHQ-9) and secondary (quality-adjusted life years) trial results. Sensitivity analyses will probe the influence of varying factors such as analysis duration, pivotal cost components, and the confidence intervals of trial outcomes. The trial’s equity impact will be assessed by analysing income quantiles and measurements of multidimensional poverty. A detailed description of the planned economic evaluation will be presented in a separate health economics protocol.

### Methods in analysis to handle protocol non-adherence and any statistical methods to handle missing data {20c}

For the management of missing data, we will employ a maximum-likelihood multiple imputation method, with a sensitivity analysis juxtaposed against the complete case dataset. The primary analyses will be conducted on an ITT basis. In addition, secondary analyses, encompassing per-protocol and CACE analyses, will be performed to evaluate the potential modification of treatment effect estimates due to diminished clinical engagement.

### Plans to give access to the full protocol, participant-level data, and statistical code {31c}

The study protocol will be publicly available on the sponsor’s website and the dataset will be available upon request to the study’s chief investigator. The statistical analysis plan will be published on Open Science and the code may be available upon request. Legal constraints in both participating countries limit the full implementation of Open Science protocols.

## Oversight and monitoring

### Composition of the coordinating centre and trial steering committee {5d}

The oversight of this trial will be facilitated by four committees: the Data Monitoring and Ethics Committee (DMEC), the Trial Steering Group (TSC), and two local Community Advisory Boards (CABs) situated in Kenya and Lebanon.

*Trial Steering Committee*: The TSC is tasked with the supervision of the trial. It will review the DMEC’s recommendations and, based on this information, suggest any pertinent amendments or actions for the trial. The TSC represents the interests of the funder and Sponsor.

*Community Advisory Boards*: The CABs are established to provide guidance to the study team, thereby enriching the research’s quality, feasibility, and visibility in the community through meaningful local engagement. The CABs are designed to elevate the research’s relevance by collaborating with community partners of varying expertise. Key responsibilities of the CABs include advising on the sustainability and long-term impact of the study, discussing adherence issues, supporting outcome monitoring, and advising on integrating the research findings into the broader healthcare system. Furthermore, they will review training materials to ascertain the sustainability of g-IPT and offer guidance on adapting it to local settings.

### Composition of the data monitoring committee, its role, and reporting structure {21a}

The DMEC comprises independent experts in the respective field. The Committee convenes roughly every 6 months (or as deemed necessary) to scrutinise recruitment, track follow-up rates, and examine serious adverse events. The DMEC serves in an advisory capacity to the TSC and holds the authority to suggest premature closure of the trial. The DMEC operates independently from the sponsor.

### Adverse event reporting and harms {22}

Adverse events (AEs) and serious adverse events (SAEs) will be documented and relayed in alignment with the study’s AE/SAE protocol. The local trial coordinator at each site will report all adverse events to the designated senior clinician for classification. Every AE will be evaluated in terms of its severity, causality, seriousness, and expectedness. All AEs will be relayed to the site’s Principal Investigator (PI), and SAEs will be communicated to the site PI, the programme Chief Investigator (CI), and the independent chair of the DMEC.

This systematic approach ensures consistent responses to AEs and SAEs in accordance with the study’s guidelines. Additionally, the National Mental Health Programme High-Risk Protocol in Lebanon and the Adverse Events Standard Operating Procedures in Kenya will be adhered to.

### Frequency and plans for auditing trial conduct {23}

The sponsor will establish the degree and nature of the required monitoring for the trial. Risk will be continually assessed, and modifications made in response. The extent of monitoring will correlate with the risks associated with the trial. An oversight and monitoring plan tailored to this study will be implemented.

### Plans for communicating important protocol amendments to relevant parties (e.g. trial participants, ethical committees) {25}

Prior to any participant recruitment, the Chief Investigator and the UK study team will ensure that the relevant research ethics committees have approved the trial protocol, participant information sheet, consent form, and other associated documents. Any modifications to the protocol and supporting documents, including approved amendments, will be documented and submitted for the necessary ethical and regulatory approvals. These amendments will not be implemented until the requisite approvals are secured.

Recruitment at local sites will only commence once all local permissions are in order and the UK study team has given the green light via email. It is incumbent upon the CI/PI or their designee at each site to ensure all future amendments obtain the essential approvals, encompassing both UK and local ethical permissions.

In the UK, the REC will receive an annual progress report (APR) within 30 days of the anniversary of its favourable opinion, and this will continue annually until the trial concludes. Additionally, the study representative at UCL in the UK will receive a progress report every 6 months. Within 90 days post-trial completion, the Chief Investigator will notify the main REC that the trial has concluded. If the trial ends prematurely, these notifications will be given within 15 days post-trial conclusion.

A summary report of the trial will be prepared by the CI for the Sponsor, which will then be relayed to the REC within a year after the trial’s conclusion.

In Lebanon, any intended deviation from the protocol must be disclosed beforehand to the USJ (Universite Saint Joseph) IRB. For instance, if there is a desire to alter the recruitment numbers or modify the project’s timeline, a justifying letter must be sent and approval must be acquired from the board.

In Kenya, an annual ethical renewal is mandated during the study duration. This renewal will be facilitated through an annual progress report, submitted to the local ERC 1 month before the yearly approval expires. The study coordinator will draft the APR for this purpose. Upon the study’s culmination, the local ERC will be apprised of its completion by the study coordinator. The annual renewal of NACOSTI and County research permits hinges on the progress report’s submission and the clearance of AE/SAE reporting by the IRB in Kenya.

### Dissemination plans {31a}

The primary medium for disseminating the study’s results will be through publications in reputable peer-reviewed journals. Additionally, these results will be integrated into presentations at conferences, seminars, and workshops. Collaborative partners will also discuss and reference the study in their respective articles and communications. The Community Advisory Boards will advise on key stakeholders in Lebanon and Kenya and strategies to reach them. To reach a wider audience, the findings will also be communicated through press releases, webinars, and informative sessions for healthcare professionals and relevant stakeholders. In Kenya, local engagement will take place at both the County and National levels and policy briefs will be produced. In Lebanon, findings will be disseminated through the design of national mental health campaigns around maternal mental health—specifically post-natal depression. This campaign will include inter-ministerial collaboration (Ministry of Communication, MoPH & others). Evidence-based and culturally adapted key messages will be tailored towards the general public throughout all local media channels and PHC networks in Lebanon. Evidence-based awareness sessions and supporting materials for the general public will be delivered as part of the PHCs’ service to their local communities.

## Discussion

PND is a pressing public health concern which impacts substantially on both maternal health and child development. While research from high-income countries has established the efficacy of IPT and group-IPT (g-IPT) for PND, its effectiveness within the LMIC context and its potential benefits for child development remain uncharted and this study will provide a rigorous evaluation of its effectiveness and cost-effectiveness compared to HQ-SC in two LMICs. The design of this study has been strengthened by the conceptual mapping and feasibility phases which have ensured appropriate adaptation of g-IPT for the contexts and populations it is being delivered to, as well as mapping and strengthening local care pathways to ensure the sustainability of the treatment if it is found to be effective and cost-effective. With this phase of the research programme complete, the RCT can proceed with reasonable confidence that the protocol detailed above is acceptable to all the sites and that recruitment, data collection, and training are feasible in all locations. The trial will also deliver data on the clinical and economic benefits of the low-cost control treatment. It is anticipated that the trial’s findings will help to inform future decision-making about policy and services both in Kenya and Lebanon and beyond.

A challenge facing this programme is the current inadequacy of perinatal mental health services at both sites. This is marked within the refugee population in the Lebanon and across the low socio-economic status population in Nairobi. The lack of specialised mental health care resources and community-based mental health services in Lebanon is a major contributor to the treatment gap (WHO-AIMS Lebanon, 2010). Local communities have been under tremendous pressure to meet the high demands, especially with the surge of displaced Syrians, stretching resources and infrastructures that were already insufficient for the hosting communities. In order to meet these needs, the Ministry of Public Health (MoPH) of Lebanon initiated the National Mental Health Programme in 2015, which initiated a 5-year Mental Health Strategy (2015–2020), currently extended until 2030. The programme’s goal is to provide vulnerable populations with comprehensive, integrated, and responsive mental health services in community-based settings [68]. Currently, in Kenya, there is no screening of mental health conditions in the primary health care facilities; most postnatal women who come to the facility for child immunisation or well-child clinic are experiencing mental health conditions, but because there is no screening happening, these women go home with their conditions undiagnosed [69]. While this presents a significant challenge to be met during this RCT, it also highlights the urgency of the trial and its relevance to a substantial public health issue affecting a very high proportion of women experiencing PND and their children brought up with limited maternal support.

Although both in Lebanon and Kenya current policy strongly supports the development of mental health provision particularly in perinatal mental health, financial crises in either or both countries could impact the development of new pathways that this research aims to create.

The ongoing economic downturn in Lebanon, exacerbated by escalating fuel prices, has made commuting to the centres a challenge for some SUMMIT patients, especially those living further away. This issue was highlighted during the feasibility phase of this research. To address this, we provided symbolic incentives during data collection periods, assisting participants in offsetting some of their transportation expenses.

Furthermore, the prevailing political environment in Lebanon, combined with the Lebanese government’s deportation policy affecting Syrian nationals, created apprehension among our Syrian participants. This may lead to sporadic attendance in the intervention groups and an increase in cases lost to follow-up in our Syrian population. To mitigate this, we have adjusted session schedules to more convenient times. Fortunately, recent relaxations in deportations and sanctions by the Lebanese government have somewhat alleviated these concerns, resulting in improved attendance and adherence to intervention sessions among the Syrian participants.

Given the current political unrest in Lebanon, particularly the bombings taking place in the South of Lebanon, and in order to safeguard the safety of the SUMMIT team, along with the patients who attend their sessions with their children, the team has prepared a contingency plan to shift sessions and data collection time points online, in case the situation deteriorates and central Beirut becomes affected.

This programme of research, particularly in Lebanon, is strongly embedded within a gender equality social initiative. It is recognised that in many countries in the Middle East, gender equality creates cultural and sometimes political challenges. The long-term success of this programme evidently depends on a political climate favourable to gender equality as a value.

While in Lebanon the control of professional psychological practitioners is strong thus ensuring high-fidelity evidence-based treatments being sustained, in Kenya professional bodies that ensure the technical competence of practitioners are less well established. We hope that this programme will serve to begin to mitigate this issue particularly around the availability of appropriately skilled staff offering services to women with PND.

In response to the COVID-19 pandemic, we have adapted our operational approach to ensure the safety of both participants and the SUMMIT team. Measures, consistent with local health guidelines, have been implemented to prevent COVID-19 transmission. In Kenya, all members of the SUMMIT team will be fully vaccinated. During all interactions, there should be strict adherence to the Ministry of Health’s protocols.

## Trial status

The trial began recruiting participants on 2 January 2023 and was scheduled to finish recruitment on 30 November 2023; however, recruitment has been faster than anticipated and our final participant was randomised on 23 October 2023. Data collection is ongoing and the anticipated end of data collection is 30 November 2024. Due to the unforeseen speed of recruitment, we have been unable to submit this protocol prior to recruitment ending. The trial is currently using version 1.1 (23.02.2023) of the protocol.

### Supplementary Information

**Additional file 1.** Trial flow diagram.

## Data Availability

Any data required to support the protocol can be supplied on request.

## Abbreviations

LMICs: Low- and middle-income countries
PND: Post-natal depression
HICs: High-income countries
NICE: National Institute for Clinical Excellence
WHO: World Health Organization
g-IPT: Group interpersonal therapy
RCT: Randomised controlled trial
CBT: Cognitive-behavioural therapy
IPT: Interpersonal therapy
BA: Behavioural activation
HQ-SC: High-quality standard care
PHCs: Primary healthcare centres
NGO: Non-governmental organisation
TAU: Treatment as usual
ACT: Acceptance and Commitment Therapy
UCL: University College London
AE: Adverse event
SAE: Serious adverse event
MDAT: Malawi Developmental Assessment Tool
PHQ-9: Patient Health Questionnaire—depression module
FCI: Family Care Indicator
CREDI: Caregiver Reported Early Development Index
GAD-7: General Anxiety Disorder-7
SCI: Sleep Condition Indicator
BISQ: Brief Infant Sleep Questionnaire—Revised Short form
LSNS-6: Lubben Social Network Scale
ICECAP-A: ICEpop CAPability measure for Adults
ICC: Intra-class correlation
REDCap: Research Electronic Data Capture
CHVs: Community Health Volunteers
GDPR: General Data Protection Regulations
GCP: Good Clinical Practice
SoPs: Standard Operating Procedures
CRFs: Case report forms
ITT: Intention-to-treat
SAP: Statistical Analysis Plan
DMEC: Data Monitoring and Ethics Committee
TSC: Trial Steering Group
CABs: Community Advisory Boards
APR: Annual progress report
REC: Research Ethics Committee
CI: Chief Investigator
PI: Principal Investigator
IRB: Institutional Review Board
ERC: Ethics Review Committee
