# Supplementary material for: Implementing and evaluating group interpersonal therapy for postnatal depression in Lebanon and Kenya—individually randomised superiority trial
Source: Trials. 2024 Mar 26;25:217. doi: 10.1186/s13063-024-08039-3 (PMC10964704; doi:10.1186/s13063-024-08039-3)
Supplement: Supplementary file 1 — Additional file 1. Trial flow diagram. [file 13063_2024_8039_MOESM1_ESM.docx]

Referral is sent back to county hospitals; the primary health care centre or private clinic and HQ-SC is put in place.

**Screening**

In Kenya, community health volunteer will identify potential participants and will complete the two Whooley Questions, if the person answers “yes” to either question they will then complete the PHQ-9.
In Lebanon, health staff members will identify potential participants and then complete the PHQ-9. In both countries, if their PHQ-9 score is 12 or more they will be referred for clinical review to assess their eligibility.

A clinician, clinical officer or nurse will meet with the participant. During this meeting the MMS (section C only) will be completed, they will then explain the purpose of the study, the process and if they agree to take part in the study, what participation would involve. They will also describe g-IPT and HS-QC. During the meeting, the potential participant is given a copy of the participant information sheet (PIS).

The screening does not meet criteria

OR

The participant chooses to opt out of taking part in the study

**Consent and baseline:** A member of the local research team will wait at least 48 hours before contacting the participant after they have received the PIS. If the participant decides they want to take part in the trial, the researcher will arrange to meet with them to take informed consent and then complete the baseline outcomes measures.

**Randomisation**

Once informed consent has been given and baseline measures completed the researcher notifies the local trial coordinator. The local trial coordinator then enters the participant’s randomisation information into the NWORTH online system that randomly assigns the participant to either of the two conditions. The participant will be informed of the allocation but the local researcher will remain blind to treatment allocation.

**188 = HS-QC**

**224 = HQ-SC + g-IPT**

**Treatment stage**

**HQ-SC:** All participant will receive two sessions of HQ-SC.

**G-IPT:** Participants randomised into this arm will go on to receive 8 sessions of g-IPT, over the course of approximately 8 weeks.

**Research follow-up**

All participants will be follow-up for 52 weeks after their first session of HQ-SC. A member of the local research team will contact the participant to complete a series of research follows up in person or over the phone (only if the participant cannot attend in person) at 8 weeks (T2), 13 weeks (T3), 24 weeks (T4), 36 weeks (T5) and 52 (T6) weeks after their first session of HQ-SC.
